# Heterosexual and Homosexual Partners Practising Unprotected Sex May Develop Allogeneic Immunity and to a Lesser Extent Tolerance

**DOI:** 10.1371/journal.pone.0007938

**Published:** 2009-11-23

**Authors:** Cherry Kingsley, Barry Peters, Kaboutar Babaahmady, Laura Pomeroy, Durdana Rahman, Robert Vaughan, Thomas Lehner

**Affiliations:** 1 Mucosal Immunology Unit, Kings College London, Guy's Hospital, London, United Kingdom; 2 Department of Infectious Diseases, Kings College London, London, United Kingdom; 3 Department of Tissue Typing, Kings College London, Guy's Hospital, London, United Kingdom; New York University School of Medicine, United States of America

## Abstract

**Background:**

Epidemiological studies suggest that allogeneic immunity may inhibit HIV-1 transmission from mother to baby and is less frequent in multiparous than uniparous women. Alloimmune responses may also be elicited during unprotected heterosexual intercourse, which is associated *ex vivo* with resistance to HIV infection.

**Methodology/Principal Findings:**

The investigation was carried out in well-defined heterosexual and homosexual monogamous partners, practising unprotected sex and a heterosexual cohort practising protected sex. Allogeneic CD4^+^ and CD8^+^ T cell proliferative responses were elicited by stimulating PBMC with the partners' irradiated monocytes and compared with 3^rd^ party unrelated monocytes, using the CFSE method. Significant increase in allogeneic proliferative responses was found in the CD4^+^ and CD8^+^ T cells to the partners' irradiated monocytes, as compared with 3^rd^ party unrelated monocytes (p≤0.001). However, a significant decrease in proliferative responses, especially of CD8^+^ T cells to the partners' compared with 3^rd^ party monocytes was consistent with tolerization, in both the heterosexual and homosexual partners (p<0.01). Examination of CD4^+^CD25^+^FoxP3^+^ regulatory T cells by flow cytometry revealed a significantly greater proportion of these cells in the homosexual than heterosexual partners practising unprotected sex (p<0.05). *Ex vivo* studies of infectivity of PBMC with HIV-1 showed significantly greater inhibition of infectivity of PBMC from heterosexual subjects practising unprotected compared with those practising protected sex (p = 0.02).

**Conclusions/Significance:**

Both heterosexual and homosexual monogamous partners practising unprotected sex develop allogeneic CD4^+^ and CD8^+^ T cell proliferative responses to the partners' unmatched cells and a minority may be tolerized. However, a greater proportion of homosexual rather than heterosexual partners developed CD4^+^CD25FoxP3^+^ regulatory T cells. These results, in addition to finding greater inhibition of HIV-1 infectivity in PBMC *ex vivo* in heterosexual partners practising unprotected, compared with those practising protected sex, suggest that allogeneic immunity may play a significant role in the immuno-pathogenesis of HIV-1 infection.

## Introduction

Allogeneic immunity is the most potent natural immune response, as is observed in rejection of foreign tissues or organ transplants. However, natural allogeneic tolerance can be equally robust, as is seen in maternal tolerance of the fetal paternal semi-allogeneic HLA. These two reciprocal mechanisms have occupied the central stage of immunology. The critical importance of mature DC in immunity and immature DC in tolerance has been well documented [1], [2]. Interaction between HLA and TCR are significantly affected by costimulatory molecules, cytokines and chemokines. Immunoregulatory CD4^+^CD25^+^FoxP3^+^ T cells (Tregs) have greatly influenced the concept of suppression of immune responses, and are known to inhibit autoimmune diseases [3]–[5] and elicit transplantation tolerance [6], [7]. In contrast, CD40L expression by CD4^+^ T cells interacts with CD40 on DC [8], B cells [9] and CD8^+^ T cells [10] and is a potent ligand inducing diverse immune responses.

The present study was based on the hypothesis that allogeneic stimulation of human monocyte derived DC may elicit in CD4^+^ T cells either immune responses, or tolerance identified by expression of CD25^+^FoxP3^+^ regulatory cells. Alloimmune responses may inhibit HIV-1 transmission, as has been documented in vertical transmission from mother to baby [11]. HIV-1 infection is more frequent in uniparous than multiparous women [12]. Furthermore, alloimmune responses elicited during unprotected heterosexual intercourse was significantly associated ex vivo with resistance to HIV-1 infection [13]. Mucosal allogeneic responses were also elicited in rhesus macaques by direct rectal or vaginal application of allogeneic PBMC [14].

The first objective of this investigation was to study allogeneic responses in homosexual and heterosexual couples practising unprotected sex and to compare these between the two cohorts. The second objective was to identify immunological criteria that differentiated those practising unprotected and protected sex and may have affected infectivity by HIV-1. Both cohorts showed significant allogeneic proliferative responses of CD4^+^ and CD8^+^ T cells stimulated by the partners' irradiated monocytes (one way MLR), compared with 3^rd^ party unrelated monocytes. A small proportion of partners' cells however, showed tolerization of CD4^+^ and CD8^+^ T cells. Examination of CD4^+^CD25^+^FoxP3^+^ T regulatory cells in the two cohorts revealed a significantly greater proportion of these cells in the homosexual than heterosexual partners and they were associated with the CD4^+^ T cell proliferative responses. These immune responses must have been elicited by the partners' HLA stimulating lymphoid cells in the rectal, vaginal and/or penile mucosa. Comparison of heterosexual partners practising unprotected with those practising protected sex showed a smaller proportion of CD4^+^ T cells derived from the protected cohort being allo-immunized. *Ex vivo* HIV-1 infectivity studies of PBMC suggest that allogeneic immunized heterosexual subjects practising unprotected sex may have acquired some protection when compared with those practising protected sex.

## Results

### Allogeneic Stimulation of CD4^+^ and CD8^+^ T Cells with DC Induces T Cell Proliferative Responses and CD4^+^CD25^+^FoxP3^+^ Regulatory T Cells

**Figure 1:**
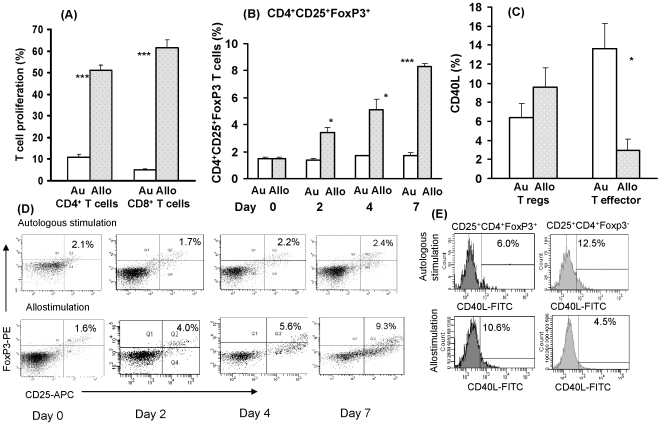
Allogeneic stimulation of T cell proliferation and CD4^+^ T regulatory cell expression *in vitro*. (A) Stimulation of CD4^+^ and CD8^+^ T cells proliferation by allogeneic DC (ratio 10∶1), (B) Comparative time-course expression of CD4^+^CD25^+^FoxP3^+^ T cells stimulated with allogeneic or autologous DC; (C) reversal of CD40L expression in CD25^+^FoxP3^+^ Treg cells and CD25^+^FoxP3^−^ T effector cells; (D) representative illustration of (B), and (E) representative illustration of (C); Au – autologous, Allo-allogeneic; *p<0.05, **p≤0.01, ***p = 0.001 in 3–4 independent experiments.

Human PBMC were stimulated with unmatched monocyte-derived DC, which differed at least by one allele, and with autologous DC controls. The CD4^+^ T cells were >95% pure and the DC expressed cell-surface CD83, CD80, CD86 and CD40 molecules (data not presented). Allogeneic stimulation of CD4^+^ or CD8^+^ T cells elicited conventional proliferative responses (Figure 1A). Allogeneic stimulation induced in addition CD4^+^ regulatory T cells. To establish optimal co-culture conditions to generate CD4^+^CD25^+^FoxP3^+^ T reg cells, preliminary studies of the ratio of DC to CD4^+^ T cells (1∶2, 1∶10, 1∶20 and 1∶100) were examined and this was shown to be at 1∶10 DC∶CD4^+^ T cell ratio (data not presented). A time course of 1–7 days revealed that co-culture of DC with T cells for 7 days resulted in a progressive increase in T regs from 1.5(±0.1)% on day 0, to 3.4(±0.4)% on day 2 (p = 0.05), 5.1(±0.8)% on day 4 (p = 0.04), reaching a maximal increase of 8.3(±0.2)% on day 7 (p = 0.001) (Figure 1B); representative illustrations of flow cytometry are shown in Figure 1D. The regulatory T cells were then examined for the expression of CD40L, which in the steady state was found in 7.6(±0.2)% CD4^+^CD25^+^FoxP3^+^ T reg cells and 28.9(±1.1)% CD4^+^CD25^+^FoxP3^−^ effector cells (not presented). Allogeneic (9.6±2.0%) compared with autologous stimulation (6.4±1.5%) of CD4^+^ T cells showed an increase in CD40L expression in T reg cells (p = 0.066), but a significant decrease in CD40L^+^ effector T cells, from 13.6±2.7 to 3.0±1.1% (p = 0.022) (Figure 1C); representative illustration is presented in Figure 1E. Thus, allogeneic stimulation of CD4^+^ T cells upregulated the CD40L^+^ Treg cells and downregulated the CD40L^+^ T effector cells.

### Putative *In Vivo* Allogeneic Immunization or Tolerization following Unprotected Homosexual Intercourse

**Figure 2:**
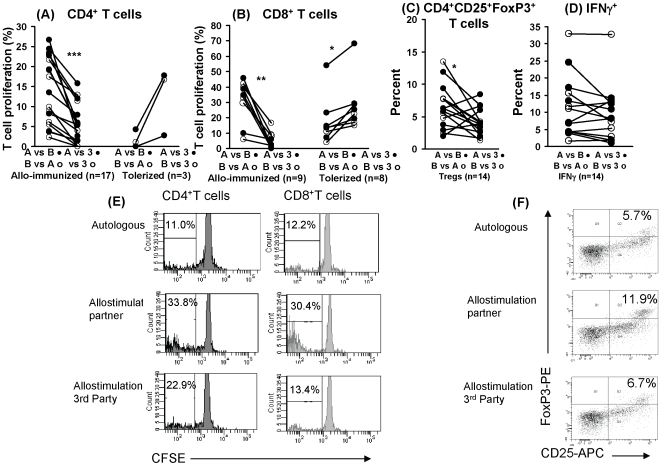
CD4^+^ T cell proliferation and regulatory cell expression in homosexual partners. Analysis of the proliferative responses of T cells isolated from homosexual subjects practising unprotected intercourse after stimulation with partner or 3rd party monocytes. Allo-immunized or tolerized (A) CD4^+^ T cells and (B) CD8^+^ T cells; 3 subjects showed no change. (C) Percentage of CD25^+^CD4^+^FoxP3^+^ T reg cells and (D) percentage of IFN-γ in CD4^+^ cells of allo-immunized subjects after stimulation with partner or 3rd party monocytes. (E) Representative illustration of CD4^+^ and CD8^+^ T cell proliferation data from an allo-immunized subject in (A) and (B), including autologous controls which were subtracted from values shown in (A) and (B). (F) Representative illustration of data in (C); *p≤0.01, **p = 0.001, ***p<0.0001.

Allogeneic stimulation *in vitro* elicited both T cell proliferative and regulatory CD4^+^ T cells. In order to see if these findings could be translated to a human model, we studied homosexual monogamous partners practising unprotected sex for at least 1 year (Table 1). These were separated by medical staff skilled in taking sexual histories into a receptive and insertive group. HLA-A, B, C, DR, DP and DQ typing showed that all partners had at least one mismatch consistent with an MLR response. The CD4^+^ and CD8^+^ T cell proliferative responses were examined between the receptive and insertive partners, as well as each against an unrelated third party monocytes. Analysis of the one way MLR showed clearly that whilst in 17 subjects there was a higher response to the partner's monocytes than those of the 3^rd^ party, in 3 subjects the reverse was observed. The different responders were separated into two groups: (1) partners showing higher MLR than those with 3^rd^ party cells were classified as putative allo-immunized and (2) the ones showing a lower MLR as putative tolerized responses. Analysis of the CD4^+^ T cells from the receptive partner stimulated by the partner's monocytes, compared with those of 3^rd^ party cells showed a significantly higher CD4^+^ T cell proliferation stimulated by the partners' than 3^rd^ party cells (p = 0.003). Similar results were found with the insertive partners' cells stimulated with the 3^rd^ party CD4^+^ T cells (p<0.0001). As there was no significant difference in the MLR between the receptive and insertive subjects (p = 0.122) we combined them (mean 13.9±sem 2.0%) and analysed against the 3^rd^ party cells (5.0±1.2%). A significant increase in CD4^+^ T cell proliferation was found in the immunized subjects (p = 0.0001; Figure 2A). There were only 3 putative tolerized partners and they showed a converse MLR response to the 3^rd^ party PBMC, which was higher (12.3±4.8%) than that found with the partners' PBMC (1.4±1.4%), but the 5% level of significance was not reached, which was probably due to the small number of subjects (Figure 2A). However, the difference between the immunized (13.9±2.0%) and tolerized (1.4±1.4%) CD4^+^ T cells was significant (p<0.0001). We have analyzed the MLR against the number of episodes of unprotected sexual intercourse over the past year by plotting each subject with reference to the allo-immune or tolerance response. None of the 3 cohorts showed any correlation between these parameters. We have similarly plotted the time of last intercourse against MLR, which also failed to show any correlation.

**Table 1:** Description of the 3 cohorts of uninfected partners practising unprotected or condom-practised sex.

Group	Sexual Practice	Gender	Number of Subjects	Mean Age (Years)
1. Unprotected sex	Homosexual	Male	20	30.85±1.88
2. Unprotected sex	Heterosexual	Female	10	24.4±1.23
		Male	10	24.2±1.3
3. Protected sex	Heterosexual	Female	9	27.3±1.1
		Male	9	29.5±1.15

A similar analysis was pursued with the CD8^+^ T cells, which showed the combined CD8^+^ T cell proliferative response (partners A and B) between the immunized partners was significantly greater (31.0±4.7%) than the corresponding responses to the 3^rd^ party PBMC (4.9±1.8%, p = 0.001) (Figure 2B). Conversely, the putative tolerized partners showed significantly lower CD8^+^ T cell proliferative responses (17.7±5.6%) than those elicited by the 3^rd^ party cells (28.1±6.0%, p = 0.004; Figure 2B). However, unlike with the CD4^+^ T cells, the proportion of immunized (31.0±4.7%), compared with tolerized CD8^+^ T cells (17.7±5.6%) failed to reach the 5% level of significance (p = 0.088). Altogether the results suggest that homosexual intercourse may elicit mostly immune CD4^+^ T cell proliferative responses (17 out of 20 partners), compared with 9 out of 20 yielding CD8^+^ T cell proliferation. A decrease in CD4^+^ T cell proliferation, consistent with tolerization was found only in 3 out of 20 partners, but was greater with CD8^+^ T cells (8 out of 20 partners, the remaining 3 showing no change).

### Putative *In Vivo* Allogeneic Immunization or Tolerization following Unprotected Heterosexual Intercourse

**Figure 3:**
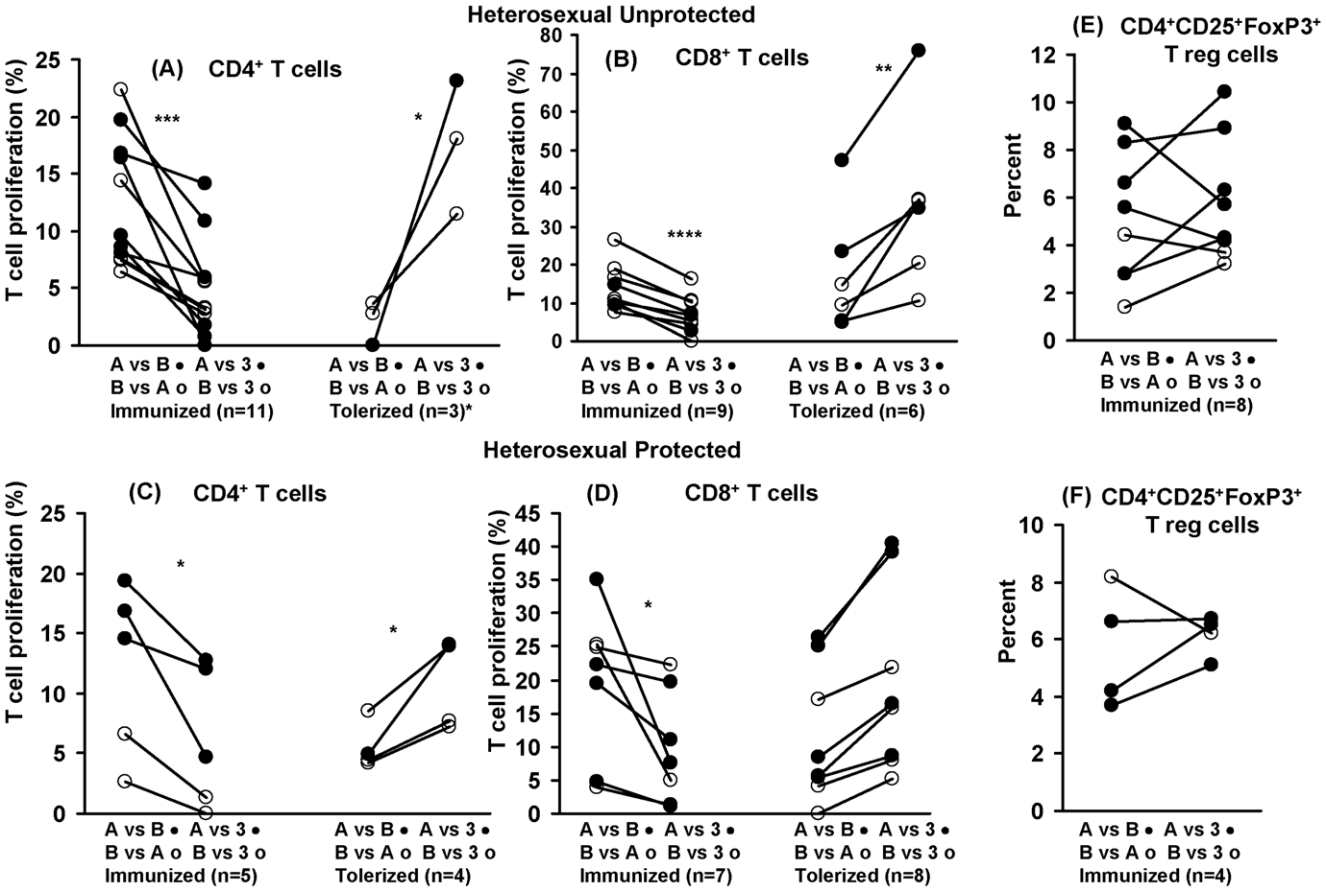
CD4^+^ T cell proliferation and regulatory cell expression in heterosexual partners practising unprotected or protected sex. Analysis of the proliferative responses of T cells isolated from heterosexual partners practising either unprotected or protected sexual intercourse; the T cells were stimulated with the partners' or 3rd party monocytes. Allo-immunization or tolerization of (A) CD4^+^ T cells and (B) CD8^+^ T cells in heterosexual subjects practising unprotected sex (5 in each showed no change); (C) and (D) in those practising protected sex; 8 and 3 showed no change, respectively. (E) and (F) Percentage of T reg cells in the two respective cohorts; *p<0.05, **p<0.01, ***p = 0.001, ****p<0.0001.

A similar analysis of PBMC from heterosexual subjects practising unprotected sex stimulated with the partners' monocytes showed a significantly higher proportion of responding cells to the partners' (12.5±1.7%) than those of 3^rd^ party cells (4.9±1.3%, p = 0.001) (Figure 3A, Figure S1A). Conversely, an increase in CD4^+^ T cell proliferation was found in the proportion responding to partners' cells (2.1±1.1), compared with those to the 3^rd^ party irradiated cells (17.6±3.4) in the putative tolerized cells (p = 0.049, Figure 3A). However, unlike in the homosexual partners, 6 of the 20 heterosexual couples showed no change (±2%) in their MLR. CD8^+^ T cells yielded comparable results but with a larger number of putatively tolerized subjects (6 instead of 3), as was also noted in the homosexual cohort. A significant increase (p<0.0001) was found in CD8^+^ T cell proliferation stimulated by the partners' cells (13.9±2.0%) compared with 3^rd^ party cells (7.1±1.6%) in the immunized couples and the converse was found in the tolerized couples (17.4±6.6% and 35.8±9.1%, respectively; p = 0.009; Figure 3B, Figure S1B). It is noteworthy that whereas the CD4^+^ T cells showed a significant increase (p = 0.0001) in the allo-immunized (12.5±1.7%) compared with the tolerized couples (2.1±1.1%), this was not found with the CD8^+^ T cells (13.9±2.0 compared with 17.4±6.6%, respectively; Figure 3A, B).

### Heterosexual Partners Practising Protected Sexual Intercourse

A further cohort of 16 heterosexual subjects (8 partners) were recruited practising condom protected sexual intercourse. Of the 16 subjects only 5 were immunized (p = 0.045), 4 tolerized (p = 0.036) and 8 showed no change in the CD4^+^ T cell proliferative response (data not presented), when stimulation with the partners' monocytes were compared with those of 3^rd^ party cells (Figure 3C, Figure S2A). The corresponding CD8^+^ T cells also showed significant immunization in 7 (Figure 3D, p = 0.045) and tolerization in 8 subjects (Figure 3D, Figure S2B, p = 0.002). However, comparison of CD4^+^ T cells proliferation in the immunized heterosexual cohort of 11/19 subjects practising unprotected sex with 5/16 practising protected sex or the tolerized cohort, as well as the CD8^+^ T cells in the corresponding cohorts failed to reach the 5% level of significance.

### CD4CD25^+^FoxP3^+^ Regulatory T Cells

CD25^+^FoxP3^+^ regulatory CD4^+^ T cells were identified by triple immunofluorescence flow cytometry with mAb to CD4, CD25 and FoxP3 antigens. A significant increase in the T regulatory cells (p = 0.013) was found in the cells from homosexual subjects, practising unprotected sex when stimulated with the partners' cells (6.6±0.9%), compared with the 3^rd^ party cells (4.0±0.5%; Figure 2C). The 2 putatively tolerized subjects could not be analyzed statistically, but showed a mean of 4.0 and 3.6%, respectively. Analysis of CD25^+^FoxP3^+^CD4^+^ T cells from the unprotected heterosexual couples showed no difference between the partners' and 3^rd^ party stimulated cells (5.1±1.0 and 5.8±0.9%, respectively (Figure 3E). Although the proportion of T regs in the tolerized couples was higher (5.1±2.1%) within the partners' cells than those of the 3^rd^ party stimulated cells (3.9±1.7%), this was not significant, possibly due to the small number (n = 3) of tolerized subjects (data not presented). Immunization was significantly more frequent in the homosexual (14/16) than hetero-sexual cohorts (8/16, p = 0.029), but no difference was found among the tolerized groups (p = 0.626). We have compared the T regulatory cells against the number of episodes of unprotected sexual intercourse for each subject in the 3 cohorts and found no correlation between these parameters.

**Figure 4:**
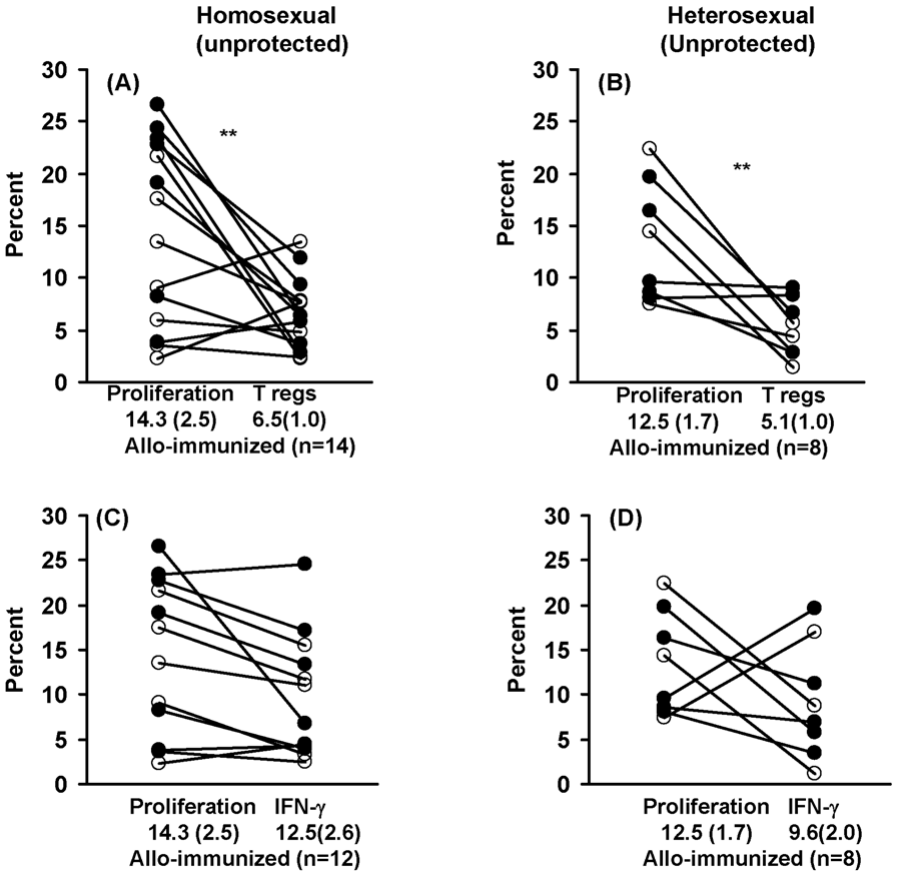
CD4^+^ T regulatory or IFN-γ producing cells related to the proliferative responses. Homosexual and heterosexual cohorts practising unprotected sex analysed for the relation (A) and (B) between CD4^+^ T cell proliferation and Treg cells; (C) and (D) CD4^+^ T cell proliferation and IFN-γ; **p≤0.01.

A significant association between the CD4^+^ T cell MLR and T regs was found in the immunized homosexual (p = 0.007) (Figure 4A) and heterosexual (p = 0.002) subjects (Figure 4B), suggesting that the MLR may be regulated by the CD4CD25FoxP3^+^ T cells. The results were also significant when CD4^+^ T regs (6.6±0.9%) were compared with CD8^+^ T cell MLR (31.0±4.7%), both for the homosexual (p = 0.001) and heterosexual cohorts (5.1±1.0% and 13.9±2%, respectively, p = 0.002). The tolerized cohorts, however, failed to show any of the above associations.

### CD4^+^ IFN-γ^+^ Th1 Cells

There was no significant difference in the percentage of IFN-γ producing CD4^+^ T cells within the PBMC population stimulated by the partners' cells compared with 3^rd^ party cells in the homosexual (Table 2.1) or heterosexual unprotected or protected partners (Table 2.2, 2.3). Furthermore, no association was observed between CD4^+^ T cell proliferation and IFN-γ producing CD4^+^ T cells either in the cells of homosexual or heterosexual unprotected or protected cohort (Table 2.4–2.6). However, a significant association was found between CD4^+^ T regs and IFN-γ producing cells in homosexual immunized subjects (p = 0.048) (Table 2.7), though this was more pronounced when stimulated with 3^rd^ party cells (p = 0.007). In heterosexual unprotected or protected subjects CD4^+^ T regs and IFN-γ producing cells failed to show a significant association (Table 2.8 and 2.9), although they showed a similar trend. The lack of statistical significance might be due to smaller number analysed (8 and 4, respectively). Attempts were made to study Th17 cells by means of IL-17 assay, but the results showed very low proportion of cells (<1%) and enhancing IL-17 with cytokines was not pursued, as this would affect the steady state of the cells. IL-4 also failed to show any changes in the 3 cohorts examined.

**Table 2:** IFN-γ expression in CD4^+^ T cells of immunized homosexual and heterosexual partners practising unprotected or protected sexual intercourse.

	IFN-γ in CD4^+^ T cells (%)		
Sexual Intercourse		Immunization	
	**No./Total**	**Partners**	**3rd Party**
1. **Homosexual** (unprotected)	17/20	11.3(2.4)	10.7(2.2)
2. **Heterosexual** (unprotected)	9/18	9.6(2.0)	10.6(2.0)
3. **Heterosexual** (protected)	4/18	8.8(1.8)	9.0(2.4)
	**(n)**	**Proliferation**	**IFN-γ**
4. Homosexual (unprotected)	(12)	14.4(2.3)	11.3(2.4)
5. Heterosexual (unprotected)	(8)	12.5(1.7)	9.6(2.0)
6. Heterosexual (protected)	(4)	8.8(1.8)	9.0(2.4)
	**(n)**	**T regs**	**IFNγ**
7. Homosexual (unprotected)	(12)	6.6(1.0)	11.3(2.4)*
8. Heterosexual (unprotected)	(8)	5.1(1.0)	9.6(2.0)
9. Heterosexual (protected)	(4)	5.7(1.0)	8.8(1.8)

The results are presented as mean (±sem); *p<0.05.

### *In vitro* HIV-1 Infectivity

**Figure 5:**
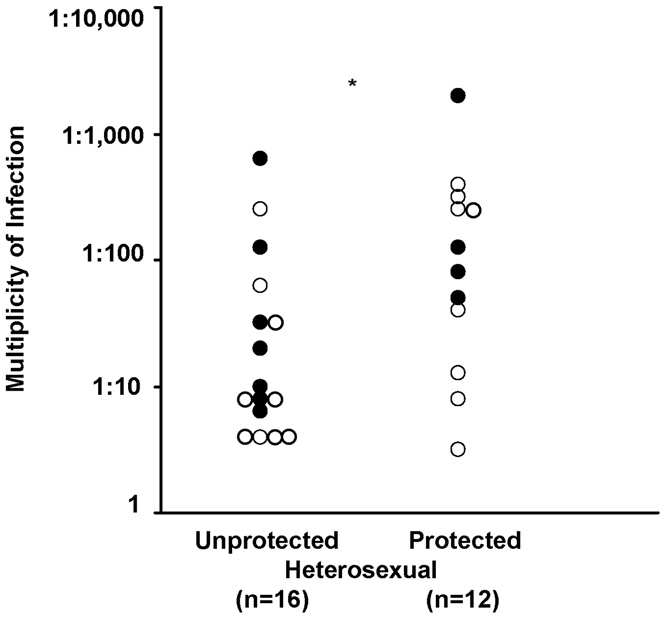
HIV-1 infectivity in heterosexual partners practising unprotected or protected sex. Multiplicity of infection of HIV-1 (BaL) required for the 50% inhibitory effect of PBMC from allo-immunized heterosexual partners (A • and B o) practising unprotected or protected sex (n = 16 and 12, respectively); *p = 0.028.

PBMC were stimulated with PHA and IL-2 for 3 days, the cells were then infected with serial dilutions of HIV-1 (BaL), cultured up to 9 days and p24 was then assayed. Analysis of the heterosexual partners practising unprotected sex (9.5 median of 50% inhibition of virus) compared with those practising protected sex (145 median) showing a significant increase in virus dilution required for the PBMC to inhibit the virus (Figure 5; p = 0.0288). The corresponding data in the homosexual subjects practising unprotected sex are not presented as a cohort practising protected sex was not available to pursue analysis with those practising unprotected sex. The results suggest that heterosexual subjects, practising unprotected sex who were allo-immunized may have acquired some protection to HIV-1 infection, as compared with those practicing protected sex. We then analysed any association between *in vitro* HIV-1 infectivity and the immunological parameters tested. No significant relationship was established with CD4^+^ T cells proliferation or with IFN-γ (Table 3.1 and 3.2). However, the proportion of CD4^+^CD25^+^FoxP3^+^ T reg cells was significantly associated with HIV infectivity in the homosexual (p = 0.011), heterosexual unprotected (p = 0.040) and protected (p = 0.030) cohorts (Table 3.3). These results suggest that T regs may affect the infectivity of PBMC in all 3 cohorts.

**Table 3:** Association between multiplicity of infection of HIV-1 (BaL) required for the 50% inhibitory effect of PBMC (Infectivity) and the proliferative response, percentage of IFNγ produced and CD25^+^FoxP3^+^ T cells (T regs) of CD4^+^ T cells; analysis by Mann Whitney test.

Homosexual (Unprotected)				Heterosexual (Unprotected)				Heterosexual (Protected)			
1)	(n)	Infectivity	CD4 prolif.	1)	(n)	Infectivity	CD4 prolif.	1)	(n)	Infectivity	CD4 prolif.
Median	(16)	62.5	15.5		(8)	8	12.0		(4)	40	16.2
		p = 0.0135				p = 0.4945				p = 0.1939	
2)		Infectivity	IFN-γ	2)		Infectivity	IFN-γ	3)		Infectivity	IFN-γ
Median	(12)	25	11.4		(8)	8	7.9		(4)	40	11.3
		p = 0.2848				p = 0.4945				p = 0.3123	
3)		Infectivity	T regs	2)		Infectivity	T regs	2)		Infectivity	T regs
Median	(14)	40	6.3		(8)	8	5.0		(4)	40	6.7
		p = 0.0030				p = 0.040				p = 0.0304	

### Analysis of HLA and CCR5 Polymorphism

There was no discernable difference in the HLA-A, B, C, DR or DQ alleles between the 3 cohorts. However, HLA-B27 was found in 5 immunized and none of the tolerized CD4^+^ T cell proliferative responses, whereas HLA B35 was identified in 2 tolerized, 2 immunized and 1 unchanged response. Although the total number of B27^+^ and B35^+^ subjects was too small for statistical analysis, the results are consistent with HLA B27 being associated with slow progression, and possibly HLA B35 with rapid progression in the development of AIDS. None of the HLA-typed individuals expressed HLA-B57 which is also associated with slow progression to AIDS. Sharing of HLA-class II was not sufficient to account for the tolerant group, as all partners showed an HLA-class II mismatch with the DR and DQ alleles, which would generate an MLR response. Heterozygous CCR5 deletions were found in 5 of the heterosexual unprotected and 4 protected subjects. Homozygous but not heterozygous deletion was found only in 1 of the homosexual subjects. There was no obvious difference in infectivity in those with or without CCR5 deletion, or in immunized and tolerized subjects.

## Discussion

Allogeneic stimulation of T cells *in vitro* elicited not only CD4^+^ and CD8^+^ T cell proliferative responses, but also CD4^+^CD25^+^FoxP3^+^ T regulatory cells. A comparative study between allogeneic and autologous stimulation of DC showed significant upregulation of CD4^+^CD25^+^FoxP3^+^ T regulatory cells by day 2 and reaching an optimum by day 7. Co-culture of allogeneic stimulated DC with autologous CD4^+^ T cells induced a greater proportion of CD4^+^CD25^+^FoxP3^+^ T reg cells expressing CD40L, whereas the reverse was found with the CD4^+^CD25^+^FoxP3^−^ effector T cells. The effect of allostimulation upregulating CD40L on T reg cells, in contrast to downregulating CD40L on CD4^+^ T effector cells is challenging and suggests an enhanced interaction of allo-stimulated T reg cells with CD40 molecules on DC, which may be re-activated thus maintaining their function. Down-regulation of CD40L on CD4^+^ T cells is consistent with skin transplant tolerance induced by neutralizing CD40L function [6].

The preliminary allogeneic studies *in vitro* were followed by potential mucosal allogeneic responses *in vivo*. Unprotected hetero-sexual intercourse has been shown in PBMC to elicit predominantly allogeneic immunization and to a limited extent tolerization.^13^ Here we have studied both heterosexual and homosexual intercourse, and have examined allogeneic immunity and putative tolerance expressed by CD4^+^ and CD8^+^ T cell proliferative responses and T regulatory cells. The results clearly demonstrate highly significant CD4^+^ and CD8^+^ T cell allogeneic immunization in partners practising unprotected homosexual (p<0.0001 and p = 0.001) or heterosexual (p = 0.001 and p<0.0001) intercourse, respectively. However, whilst tolerization of CD4^+^ T cells was found only in 3/20 homosexual and 3/19 heterosexual partners, the CD8^+^ T cells were tolerized in 8/20 homosexual partners (p = 0.004) and in 6/20 heterosexual subjects (p<0.009). Altogether the CD8^+^ T cell responses were higher than those of CD4^+^ T cells, and CD8^+^ T cells were more likely to be tolerized than CD4^+^ T cells in both types of unprotected sexual activities. The mechanism governing these findings will need to be explored. As there was an HLA-class II mismatch of the DR and DQ alleles, which would generate MLR responses, sharing of HLA-class II would not account for the tolerance.

Heterosexual subjects practising condom-protected sex were then analysed and although they also showed significant increase in CD4^+^ (p = 0.045) and CD8^+^ (p = 0.045) T cell proliferation, these were of considerably lower order of significance than those recorded in the unprotected cohorts (p = 0.001 and p<0.0001, respectively). The proportion of CD4^+^ T cells in the allogeneic immunized protected partners was also smaller (5/16) than those in the unprotected partners (11/19). The tolerized subjects showed significant increase in CD4^+^ T cell proliferation (p = 0.036), unlike those in the unprotected cohort, but the tolerized CD8^+^ T cells showed comparable levels of significance in the 2 cohorts.

To interpret the effect of unprotected rectal and vaginal intercourse on allogeneic immunity and tolerance we studied CD4^+^CD25^+^ FoxP3^+^ regulatory T cells [15]. These were significantly increased in the allo-immunized homosexual (p = 0.013) but not hetero-sexual cohorts (p = 0.440). The homosexual cohort (14/16) also showed a significantly higher proportion of T regs (p = 0.029) than the heterosexual cohort (8/16). The increased propensity of T reg cells being elicited through the rectal mucosa in homosexual men, in contrast to the cervico-vaginal mucosa in heterosexual women is an intriguing finding. This might be related to the involvement of two diverse mucosal tissues; the fragile single cell columnar epithelium of the rectum lacking Langerhans cells, compared with the stratified squamous vaginal epithelium with large number of Langerhans cells.

The overall aims were to study the 4 types of T helper cells; Th1 (IFN-γ), Th2 (IL-4 and IL-10), Th17 (IL-17) and T regulatory cells. Surprisingly IFN-γ showed no significant changes in the allogeneic immunized or tolerized homosexual or heterosexual partners and there was no significant association between IFN-γ and CD4^+^ T cell proliferation. This was found both in CD4+ T cells by flow cytometry and testing CD4^+^ T cell culture supernatants by the Luminex bead technique (results not presented). IL-4 and IL-10 assays showed very low concentrations, suggesting that Th2 cells do not play a major role. Th17 cells showed very low levels (<1%), which were not amenable to analysis as reported by others, without enhancing IL-17 expression with cytokines, such as IL-1β and IL-6 [16]. CD8^+^ T reg cells were not studied, but CD4^+^ T regs may have inhibited CD8^+^ T cell proliferative responses, as the combined homosexual and heterosexual tolerized cohorts showed an inverse correlation between the two types of cells, though the 5% level of significance has not been reached (r = 0.553, p = 0.062).

Mucosal allogeneic immunization and tolerance has received limited attention, though the importance in mucosal infections, especially of the genital tract, is evident. Ejaculates contain CD4-positive T cells, macrophages, neutrophils, immature germ cells [17], epithelial cells that express HLA antigens, and cell-free HLA antigens [18]. Furthermore, HIV-1 contains host HLA class I and II antigens [19], [20]. Multiparous females may be allo-immunized by the paternal semi-allogeneic fetus and they are less prone to HIV-1 infections than uniparous or non-parous females [12]. This has also been observed in multiparous female macaques [14]. Both rectal and vaginal exposure to allogeneic antigens during homosexual and heterosexual intercourse, respectively may elicit immunity and to a lesser extent tolerance to the monogamous partners demonstrated in this paper but the effect of multiple partners in a general population will need to be explored. However, a cohort of sero-negative female sex workers, exposed to multiple partners in Cote d'Avoire showed suppression of alloimmune responses [21].

Overall it is noteworthy that unlike in heterosexual unprotected partners, in whom 11/19 showed evidence of significant increase in MLR of CD4^+^ T cells, 3/19 a decrease and 5/19 showed no change, the corresponding data for the protected partners were 4/16, 4/16 and 8/16, respectively. The higher proportion of allogeneic CD4^+^ T cell immunized and smaller number of unchanged MLR that were observed among the unprotected compared with protected heterosexual cohorts, is consistent with increased immune activation in those practising unprotected sex. However, this was not found in the CD8^+^ T cells. Furthermore, a larger proportion of CD4^+^ T reg cells was found in the allogeneic immunized homosexual partners (14/16–88%), compared with the heterosexual partners (8/16–50%; p = 0.029), both cohorts practising unprotected sex.This was not found in heterosexual partners practising protected sex.

*Ex vivo* HIV-1 infectivity studies suggest that allo-immunized heterosexual subjects practising unprotected sex may have acquired some protection to HIV-1 infection, as a significantly lower dilution of virus was inhibited by their PBMC, compared with those practicing protected sex (p = 0.028). A similar analysis of homosexual partners practising unprotected sex could not be pursued, as homosexual partners practising protected sex for a period over 1 year could not be recruited with any confidence. We then analysed any association between *in vitro* HIV-1 infectivity and the immunological parameters tested in the 3 cohorts. HIV-1 infectivity showed significant association with the T reg cells in all 3 cohorts (p<0.05 or p<0.01), but not with CD4^+^ T cell proliferation or IFN-γ production. As T reg cells were significantly associated with CD4^+^ T cell proliferation both in homosexual (p = 0.015) and heterosexual (p = 0.010) unprotected cohorts, the data support the concept that T reg cells play a regulatory role in allogeneic stimulation and HIV-1 infectivity of CD4^+^ T cells. We have not studied further the mechanism involved, but the T reg cells may inhibit CD4^+^ T cell activation and thereby infectivity of these cells. Indeed, there is evidence that allogeneic mediated T reg cells are efficient in suppressing transplantation responses [22]. Restriction factors such as the innate anti-HIV-1 APOBEC3G is upregulated on allo-stimulation and may be involved in inhibition of HIV-1 [23].

## Materials and Methods

### Recruitment of Volunteers

Permission for this investigation was granted and approved by the Kings College Research Ethics Committee. Informed written consent was obtained from all participants. Recruitment was begun initially using a wide number of avenues, including mass-email requests through our host institutions of Guys and St Thomas' Hospitals and Kings College London, approaching asymptomatic patients seeking screening at sexually transmitted disease (STD) clinics and from members of the public who responded to advertisements. As no individuals from STD clinics met the criteria, we stopped using this avenue early on. Volunteers were excluded if they had a history of sexually transmitted or major infectious disease, at least a year prior to entry into the study. Patients were excluded if they were pregnant, or had been pregnant within 12 months of entry into the study. Patients were also excluded if they had any recent (within 12 months) severe illness, or if they had any chronic condition thought possibly to influence the study findings (eg diabetes, chronic viral hepatitis, conditions requiring steroid usage or chronic therapies, etc). This screening excluded patients who had blood transfusion within the past 12 months. We have not dealt in more depth with previous blood transfusions, as whole blood which may induce alloimmune responses has not been used in the UK over the past 11 years. As the mean age of our heterosexual “unprotected” cohort was 24.3±1.2 and the homosexual cohort 30.8±1.8, it is unlikely that a significant number would have had blood transfusions and of those that might have been transfused only about 2% develop alloimmune response. Subjects known or suspected to be HIV antibody positive were excluded and were only included if they were low risk for HIV infection or if they had a negative HIV antibody test within the previous 12 months. We enrolled three cohorts, all of which were monogamous, defined as partners that had no other sexual partners in terms of oral, anal or vaginal sex, whether protected or unprotected by means of barrier methods for the 12 months prior to enrollment in the study and for the duration of their participation in the study.

#### Group 1

Homosexual monogamous men practising unprotected sexual intercourse (10 couples, n = 20); these were men having sex with men (MSM). The criteria for entry to this group were men having unprotected penetrative anal intercourse with the same male partner for at least the past 12 months. The partners in this group were only included if there was a clear history of them having had unprotected anal sex within this relationship, and no unprotected oral or anal sex with other partners within the previous 12 months. In addition, within this group, we recorded whether the individuals practised predominantly receptive (passive) intercourse, predominantly insertive (active), or a roughly equal mixture of both.

#### Group 2

Heterosexual monogamous partners practising unprotected sexual intercourse (10 couples, n = 20). The criteria for this group were that the couples were in a monogamous heterosexual relationship, otherwise the criteria were the same as for the MSM group.

#### Group 3

Heterosexual monogamous partners practising protected sexual intercourse (9 couples, n = 18). The criteria for this group were that the couples were in a monogamous heterosexual relationship, but that any act of sexual intercourse over the previous 12 months, including oral sexual intercourse, was practised using barrier contraception.

The sexual history for all patients for all groups was taken by nurses experienced in sexual health and sexual history taking. Great care was taken, using open questioning, recording in private, and assurance of anonymity, in order to capture an accurate history. A detailed history of number, type and timings of intercourse were entered onto a structured written questionnaire for all subjects. This enabled data, such as the interval between the last intercourse and the time when the blood specimen was taken for this investigation to be evaluated.

### Isolation of Peripheral Blood Mononuclear Cells CD14^+^ Monocytes and Preparation of Immature DC

Approximately 40 mls of blood was collected from each participant into EDTA filled tubes. Peripheral blood mononuclear cells (PBMC) were isolated by centrifugation of blood on a Ficoll-Hypaque density gradient (Amersham Biosciences, Little Chalfont, Bucks, UK). CD14^+^ monocytes were enriched by depletion of CD14^-^ cells using a Monocyte Isolation Kit (MACS; Miltenyi Biotec). The purity of isolated monocytes was consistently greater than 90% when analyzed by flow cytometry with antibodies to CD14 (BD Biosciences). CD14^+^ monocytes were cultured in GM-CSF (1000 U/ml) and IL-4 (40 U/ml) supplemented medium for 4–5 days to differentiate into immature dendritic cells.

### Mixed Lymphocyte Response (MLR)

To examine whether unprotected sex resulted in allo-immunization or allo-tolerization, CFSE labelled PBMCs (responders), made up to 2×10^5^/ml in supplemented RPMI culture medium, containing 200 mMol glutamine, 100 µg/ml penicillin and streptomycin and 10% FBS, from each partner were co-cultured in 96 well plates. One way MLR were set up with autologous, allogeneic or unrelated (third party) monocytes (stimulators), made up to 1×10^6^/ml in supplemented RPMI culture medium. The cultures were incubated at 37°C for 7 days. At day 7 day supernatants from the cultures were harvested for cytokine analysis. Repeated tests with the cells from the same subjects stimulated with the partner's monocytes compared with 3^rd^ party cells showed differences in percentages but the pattern of response remained the same with reference to 3^rd^ party cells.

For intracellular cytokine and CD25^+^CD4^+^FoxP3^+^ regulatory T cell analysis, unstained PBMCs (responder cells) were co cultured with autologous, allogeneic or unrelated (third party) monocytes (stimulators) in 96 well plates and incubated at 37°C for 7 days.

For in vitro analysis of T cell proliferation to alloantigens, CFSE labelled PBMCs (responders), made up to 2×10^5^/ml in supplemented RPMI culture medium, were co-cultured in 96 well plates with 1×10^6^/ml autologous or allogeneic immature DC. The cultures were incubated at 37°C for 7 days.

### CFSE Labelling of PBMC and Flow Cytometric Analysis to Determine the Proliferative Responses of CFSE Labelled CD4^+^ and CD8^+^ T Subsets

PBMCs were suspended in pre-warmed PBS supplemented with 0.1% BSA at a final concentration of 1×10^6^ cells/ml. Aliquots of 2 µl/ml of 0.05 mM of CFSE (Invitrogen) were added to make a final working concentration of 1 uM and the cells were incubated for 10 minutes at 37°C. Staining was quenched by adding 5 volumes of ice cold RPMI to the cells, and then by incubation for 5 minutes on ice. The cells were then washed 3 times with RPMI and counted.

To determine the proliferative responses of both CD4^+^ and CD8^+^T cells after 7 days of allostimulation, CFSE labelled cells were surface stained with antibodies to CD4 (APC-CY7) (BDBiosciences) and CD8 (APC) (BD Biosciences) at a 1∶100 dilution for 30 minutes at 4°C. After washing in PBS, cells were analysed by flow cytometry using a BD FACSCanto™ II, using the software DIVA.

### Intracellular Cytokine Staining for IFN-γ and IL-4 in CD4^+^ T Cells

To determine intracellular cytokine production, cultured cells were stimulated with 5 ng/ml PMA (Sigma), 0.5 µg/ml ionomycin (Sigma) and 0.7 µg/ml golgi stop (BD Biosciences) for 4 hours at 37°C. After washing with PBS, the cells were surface stained for 20 minutes at room temperature with 5 µl of an antibody to CD4 (APC) (BD biosciences) and 0.4 µg/ml of Live/Dead Fixable Near-IR (Invitrogen) to distinguish between dead and live cells. After washing twice with PBS, the cells were fixed with 100 µl/well IC fixation solution (eBioscience) and incubated for 20 minutes at room temperature. Cells were washed twice with 1x permeabilization buffer (eBiosciences). For intracellular staining, cells were incubated with 5 µl/well of IFN-γ (FITC) and 5 ul of IL-4 (PE) (BD Biosciences) for 20 minutes at room temperature. After washing twice in 1x permeabilization buffer cells were analysed by flow cytometry as described above.

### Flow Cytometric Detection of CD4^+^CD25^+^FoxP3^+^ Regulatory T Cells

For cell surface staining, the cells were incubated with 5 µl of conjugated antibodies to CD4 (FITC) (BD Biosciences), CD25 (APC) (eBiosciences) for 30 minutes at 4°C.

For intracellular staining for the regulatory cell marker FoxP3, cells were washed in cold PBS, and 1 ml of freshly prepared Fixation/Permeabilization buffer (eBiosciences) was added to the cells, which were then vortexed and incubated for 30 minutes at 4°C. After washing twice with 2 ml 1X permeabilization buffer, cells were blocked using 2% normal rat serum for 15 minutes at room temperature. After the blocking step, cells were incubated with 10 µl of either anti-human FoxP3 (PCH101) or rat IgG2a isotype control for 30 minutes at 4°C. After washing with 1 ml of 1X permeabilization buffer, cells were analysed by flow cytometry as described above.

### Flow Cytometric Analysis of Cell Surface CD40L Expression on Regulatory and Effector T Cells after Co-Culture with Allogeneic or Autologous Immature DC

CD4^+^ T cells (2×10^6^/ml) were cultured with allogeneic or autologous immature DC (2×10^5^/ml) for 7 days in 96 well plates at 37°C. For CD40L staining or isotype control staining (IgG1, BD Biosciences), Golgistop (0.7 µl/ml, BD Biosciences) was added 10 hours before termination of the co culture. Cells were then stained and analysed by flow cytometry as described above. To differentiate between CD4^+^ Tregs and CD4^+^ effector T cells CD4^+^CD25^+^ T cells were gated according to the presence and absence of FoxP3 expression, respectively.

### HIV Infectivity of PBMCs

To test HIV-1 infection, PBMCs were activated with 10 µg/mL of PHA (phytohaemagglutinin, Sigma) in supplemented RPMI for 3 days then washed with medium and cultured in supplemented RPMI with 20 IU interleukin 2 (Schiaparelli Biosystems BV, Woerden, Netherlands) overnight. Serial dilutions of primary HIV-1 strain BaL (CCR5-binding strain) were prepared with a starting concentration of 20 ng/100 µl of p24. Aliquots of 0·6×10^6^ cells were infected with serial dilutions of 1∶10^−1^ to 1∶10^−4^ multiplicity of HIV-1 strains for 3 h. The cells were washed three times with medium and cultured in triplicates at 1×10^5^ per well in 96-well culture plates. Every 2 days, 100 µL of culture supernatant was replaced with 100 µL of medium supplemented with 20 IU interleukin 2. On day 8, the cell-free culture supernatant was assayed, with HIV-1 p24 Antigen ELISA (ZeptoMetrix Corporation, New York). The infection of human cells with HIV-BaL strain was presented as the multiplicity of infection which inhibits 50% of the virus in PBMC.

### HLA Typing and CCR5 Polymorphism

DNA was extracted from PBMC using DNA isolation kit (Promega, Southampton, UK) according to the manufacturer's instruction. The purified DNA was stored at −20°C. HLA class I and II typing for HLA-A, -B, -C, -DRB and –DQB1 was carried out by the PCR technique with sequence specific primers; the primer sequences used have been previously reported. [24], [25] PBMC showing a mismatch at more than one allele was used for allostimulation. The Δ32 CCR5 polymorphism was determined by modified PCR with sequence specific primers, with the primer 5′-TCA TCA TCC TCC TGA CAA TCG- 3′ and the antisense primer 5′ –CCA GCC CCA AGT TGA CTA TC-3′. [26] The PCR products were separated on a 2% agarose gel to maximise the separation between the wildtype (262 bp) and deletion (230 bp) products.

### Statistical Analysis

For parametric data the results were expressed as mean (±sem) and student's paired or that for two means was used. For non-parametric data the Mann-Whitney test was used. The Chi square test was applied for analysis of associations, and Fisher exact test for cells with small frequencies. Inter-group analyses between the 3 cohorts using Student's t test for two means mostly showed no significant differences as it was apparent that there were two populations – putative allogeneic and tolerogenic – that yielded opposite responses when stimulated with the partner's monocytes and compared with those of the 3^rd^ party cells (data not presented).

## Supporting Information

Figure S1 Representative illustrations of CD4^+^ and CD8^+^ T cell proliferative data in an immunized and tolerized subject shown in Figures 3(A) and (B) of heterosexual subjects practising unprotected sex; included are autologous controls which were subtracted from the values shown in Figures 3(A) and (B).
(0.03 MB PDF)

Figure S2 Representative illustration of CD4^+^ and CD8^+^ T cell proliferative data in an immunized and tolerized subject shown in Figures 3(C) and (D) from a heterosexual subjects practising protected sex; included are autologous controls which were subtracted from the values shown in Figures 3(C) and (D).
(0.03 MB PDF)
