# Supplementary material for: Heterosexual and Homosexual Partners Practising Unprotected Sex May Develop Allogeneic Immunity and to a Lesser Extent Tolerance
Source: PLoS One. 2009 Nov 23;4(11):e7938. doi: 10.1371/journal.pone.0007938 (PMC2775923; doi:10.1371/journal.pone.0007938)

**Supplementary figure 1**

**Representative illustrations of CD4$^+$ and CD8$^+$ T cell proliferative data in an immunized and tolerized subject shown in Figures 3(A) and (B) of heterosexual subjects practising unprotected sex; included are autologous controls which were subtracted from the values shown in Figures 3(A) and (B)**

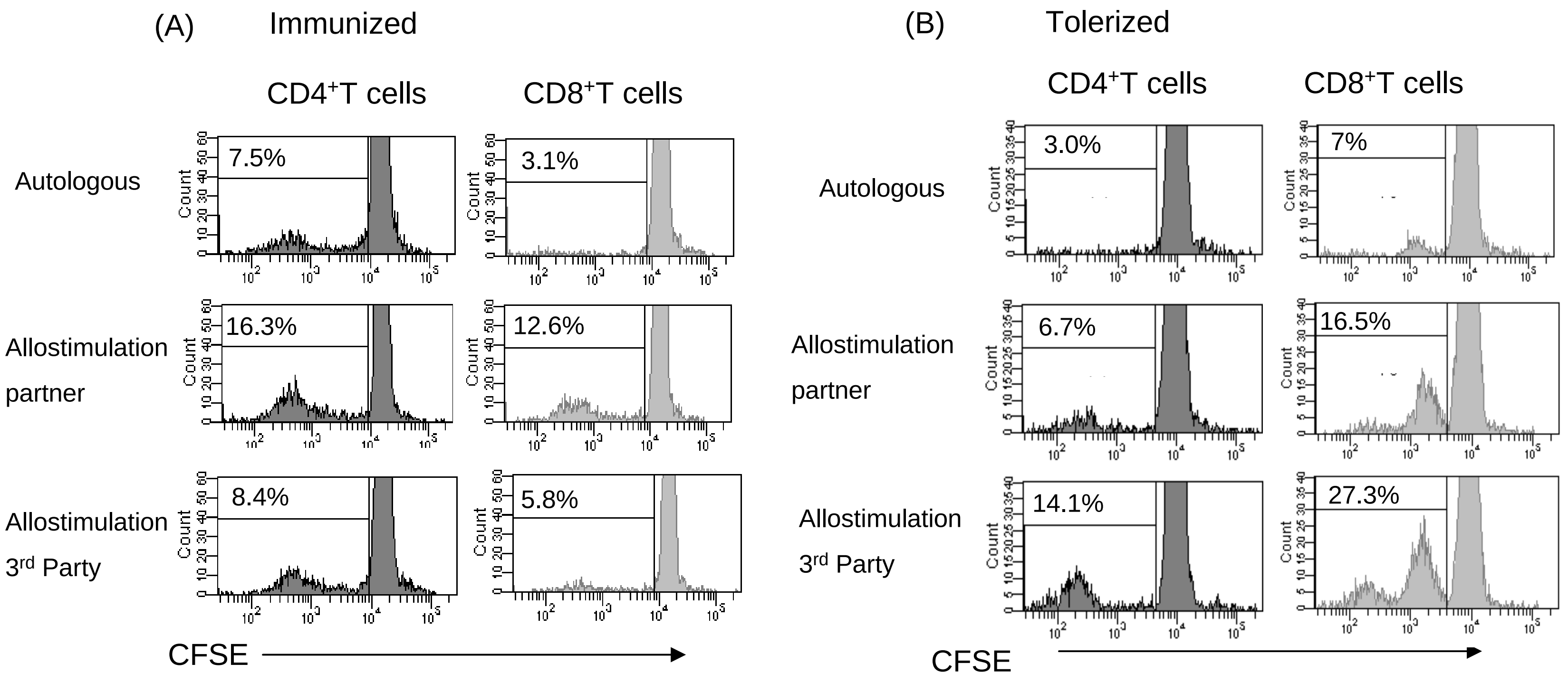

Supplement: Figure S1 — Representative illustrations of CD4+ and CD8+ T cell proliferative data in an immunized and tolerized subject shown in Figures 3(A) and (B) of heterosexual subjects practising unprotected sex; included are autologous controls which were subtracted from the values shown in Figures 3(A) and (B). (0.03 MB PDF) [file pone.0007938.s001.pdf]
