# Supplementary material for: Heterosexual and Homosexual Partners Practising Unprotected Sex May Develop Allogeneic Immunity and to a Lesser Extent Tolerance
Source: PLoS One. 2009 Nov 23;4(11):e7938. doi: 10.1371/journal.pone.0007938 (PMC2775923; doi:10.1371/journal.pone.0007938)

Supplementary Figure 2

Representative illustration of $CD4^+$ and $CD8^+$ T cell proliferative data in an immunized and tolerized subject shown in Figures 3(C) and (D) from a heterosexual subjects practising protected sex; included are autologous controls which were subtracted from the values shown in Figures 3(C) and (D).

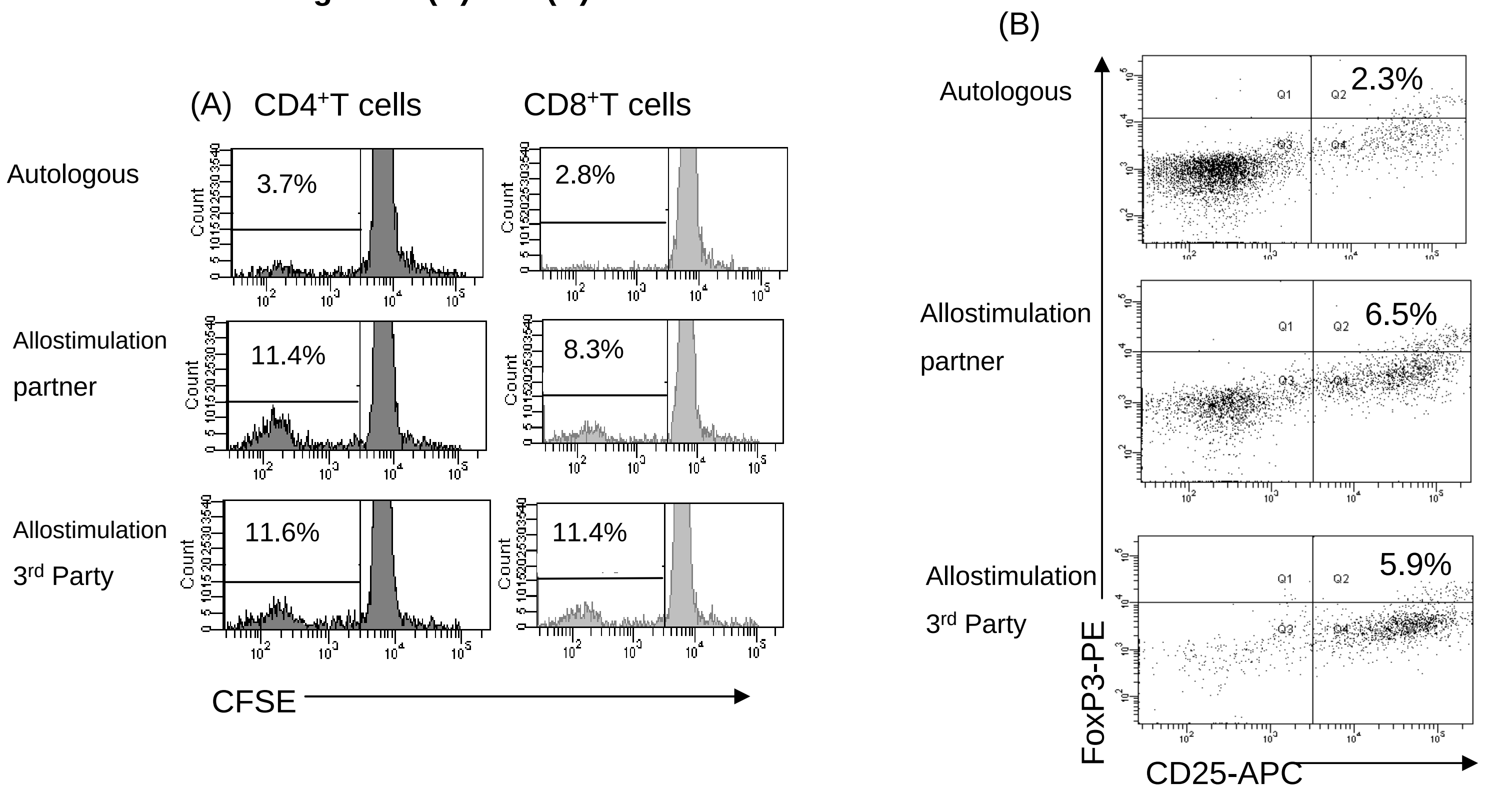

Supplement: Figure S2 — Representative illustration of CD4+ and CD8+ T cell proliferative data in an immunized and tolerized subject shown in Figures 3(C) and (D) from a heterosexual subjects practising protected sex; included are autologous controls which were subtracted from the values shown in Figures 3(C) and (D). (0.03 MB PDF) [file pone.0007938.s002.pdf]
